# Tomographic Ultrasound Imaging to Control the Placement of Tension-Free Transobturator Tape in Female Urinary Stress Incontinence

**DOI:** 10.1155/2016/6495858

**Published:** 2016-08-16

**Authors:** Charlotte M. Gräf, Tomas Kupec, Elmar Stickeler, Tamme W. Goecke, Ivo Meinhold-Heerlein, Laila Najjari

**Affiliations:** Department of Gynecology and Obstetrics, University Hospital RWTH Aachen, Pauwelsstraße 30, 52074 Aachen, Germany

## Abstract

*Purpose*. The objective was to evaluate, by means of tomographic ultrasound imaging (TUI), the reliability of a novel approach for determining the position of the implanted tension-free transobturator tape (TOT). Furthermore, we analyzed the association between the position of the tape at rest and the subjective cure in stress incontinent women.* Methods*. This retrospective pilot study consists of 32 stress incontinent women, who underwent TOT procedure and routine sonographic control at day 1 postoperatively and at follow-up visit. TUI was applied on the resulting 4D volumes, thereby delivering 9 axial slices with a 4 mm interslice distance starting at the meatus urethrae internus in caudal direction. The reliability of the approach was tested by two examiners. Postoperative and follow-up ultrasound parameters of uncured and cured patients were analyzed.* Result*s. Measurements of the position of the TOT demonstrated high intraclass correlation coefficients. We found minor differences between sonographic parameters at day 1 postoperatively and at follow-up after a median period of 321 days. In cured patients, the position of the tape was measured in a more caudal position than in uncured patients.* Conclusions*. TUI can be a reliable method for determining the position of the tape. Further studies are needed to evaluate whether the postoperatively determined position can be used as an indicator of future subjective cure.

## 1. Introduction

Suburethral tapes have become the gold standard for treating stress urinary incontinence [1]. Multiple studies [2–9] have determined the position of the tape as one relevant factor impacting the surgical outcome. Some works, however, negate such relevance [10, 11]. Various approaches for measuring the position of the tape by means of ultrasound have been proposed in the literature. These ultrasound parameters comprise, among others, the tape-symphysis pubis angle and distance [2, 11, 12] and the position of the tape in relation to the urethral length measured on midsagittal plane [2, 4–8, 11].

In this work, we introduce a fast and convenient new approach for determining the position of the tape by exploiting the advantages of the sonographic cross-sectional imaging, tomographic ultrasound imaging (TUI). One advantage of this technique is that we do not require measurements that rely on the definition of coordinate systems, which is known to be a complex task [13].

With this study we aim at evaluating the feasibility and accuracy of TUI for determining the position of implanted tension-free transobturator tape (TOT). Furthermore, we investigate the association between the position of the tape at rest and the subjective cure in stress incontinent women.

## 2. Methods

This study relies on a retrospective trial design. 91 women were eligible for the study with clinically and urodynamically proved stress urinary incontinence. The patients underwent TOT insertion with a polyvinylidene fluoride (PVDF) tape at the Department of Urogynecology of our University Hospital between January 2010 and November 2014.

Incomplete ultrasonographic data at day one postoperatively and at the follow-up examination, as well as incomplete follow-up data, were selected as exclusion criteria.

Clinical parameters were chosen from medical records. As routine, the patients answered, both preoperatively and postoperatively, the International Consultation on Incontinence Modular Questionnaire-short form (ICIQ-SF). The patient was considered subjectively cured if she (1) was stress continent, (2) had no obstructive voiding, and (3) had no* de novo* urgency at the postoperative and follow-up examination.

Patients underwent perineal ultrasound evaluation at day one postoperatively and at the follow-up examination after a period of at least 3 months.

**Figure 1:**
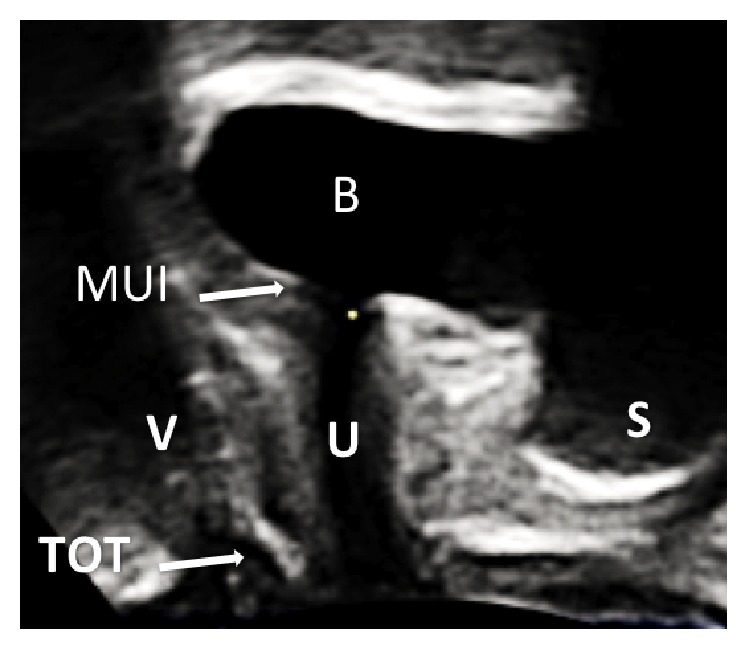
Perineal ultrasound image on midsagittal plane from a patient at rest with a tension-free transobturator tape (TOT). The positions of symphysis pubis (S), bladder (B), meatus urethrae internus (MUI), urethra (U), and vagina (V) are indicated.

These ultrasonographic investigations were conducted in a routine manner and performed by one single examiner qualified according to the DEGUM II standard. The patients were in supine position with a bladder filling of approximately 300 mL. An E8 Voluson ultrasound system (GE Healthcare Ultrasound, Zipf, Austria) was used with a perineal probe (3.5–5 MHz) placed with minimal pressure on the perineum. With an acquisition angle of 70 degrees, the image illustrated symphysis pubis, bladder, urethra, and vagina as shown in Figure 1. 4D ultrasound was then performed while the patients were instructed to rest, strain, perform the Valsalva maneuver, and cough strongly. The resulting volumes were stored for later evaluations.

**Figure 2:**
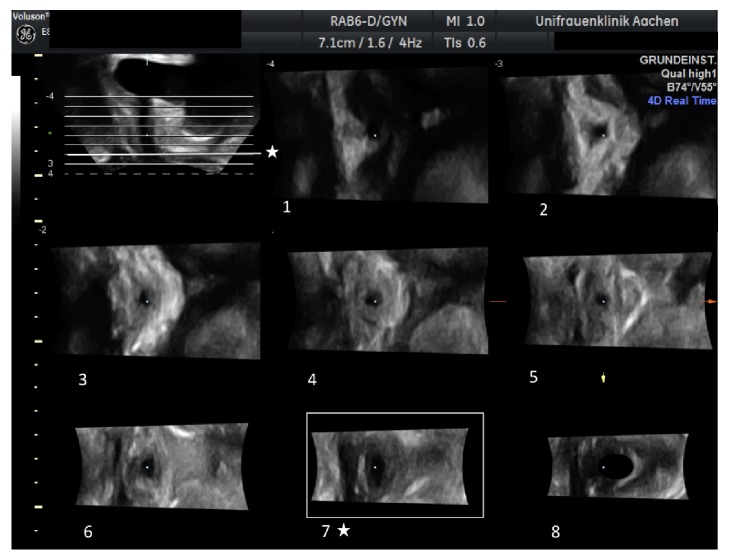
Using tomographic ultrasound imaging (TUI), 9 parallel slices are obtained (upper left figure showing the midsagittal view). The uppermost slice is placed at the meatus urethrae internus (MUI). In caudal direction eight further slices are obtained with a 4 mm interslice distance. The line marked with the symbol ☆ refers to slice number 7. This slice is shown in Subfigure 7 on the axial plane. Here, the major part of the tape is seen dorsal to the urethra.

TUI was performed on the 4D volumes obtained at rest using the 4D View (GE Healthcare) software. The uppermost slice was placed, perpendicular to the urethra, at the position of the MUI at rest. In caudal direction 8 further slices were obtained with a 4 mm interslice distance as shown in Figure 2. This distance was selected based on earlier clinical experiences, as we had not found tapes positioned outside this 32 mm range starting at the MUI.

**Figure 3:**
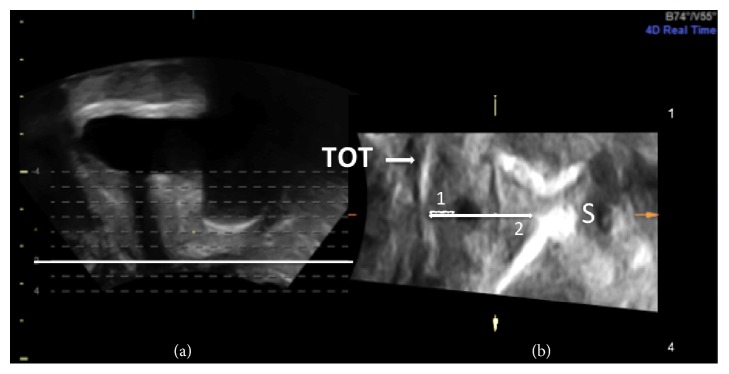
As already shown in Figure 2, tomographic ultrasound imaging (TUI) on midsagittal plane provides a set of slices (a). Here, the bold highlighted slice is represented on axial plane in (b). This view facilitates the accurate location of the tape and the measurement of distances. In particular, the image illustrates the distance between the anterior margin of the tape and the longitudinal smooth muscle (LSM) complex of the urethra (1) and the distance between the anterior margin of the tape and the inferior margin of the symphysis pubis (2).

Three sonographic parameters that describe the position of the tape were considered: first, the slice number where the tape was found dorsal to the urethra on the axial plane, thus, reflecting the craniocaudal position as shown in Figure 2, second, the distance between the anterior margin of the tape and the longitudinal smooth muscle (LSM) complex of the urethra (TUD) as introduced by Kociszewski et al. [6], and third, the distance between the anterior margin of the tape and the inferoposterior symphyseal margin (TSD). Both distances were determined on the axial plane as illustrated in Figure 3.

Two complete measurements of the sonographic parameters were performed, each by one single examiner without the knowledge of each other's results.

A DynaMesh SIS (*Dahlhausen*) tape made of PVDF was used. The surgeon applied a modified outside-in technique, which was originally introduced by Delorme et al. [14]. In general anesthesia, a perpendicular vaginal incision was made 0.5 cm below the external urethral orifice after injecting a single shot antibiotic and emptying the bladder. Afterward, a paraurethral tunnel was dissected with an angle of 45 degrees to the sagittal plane towards the obturator membrane. Next, an incision was made 2 cm above and 4 cm lateral to the meatus urethrae externus and then the TOT was inserted using the helix. The same procedure was followed on the other side. After filling the bladder, the catheter was removed. A stress test was performed by softly pressing suprasymphyseally. To optimize the tension-free position of the tape, a urethral catheter of 18 Charrière was entered and the tape was slightly adjusted. Finally, a urethral catheter of 14 Charrière was inserted and a vaginal tamponade was applied. The catheter and the tamponade were removed after 24 hours.

Statistical analysis was conducted using SPSS Version 22. Normal distribution was tested using Shapiro-Wilk test and was rejected in some cases. Hence, the Wilcoxon-signed-rank test was applied for paired data, while for independent groups the Mann-Whitney* U* test was used instead. For dichotomous samples, Fisher's exact test was applied.

To evaluate the interrater reliability of the sonographic parameters, the intraclass correlation coefficients (ICC) (two-way random, single measurements, and absolute agreement) were determined.

To assess the relation between tape position and outcome, multivariable logistic regression (backward stepwise method) was performed, yielding adjusted odds ratio (OR) with 95% confidence interval (CI).

A significance level of 0.05 was used.

This study was performed according to the Declaration of Helsinki and approved by the local ethics commission (reference number EK085/11).

## 3. Results

A total of 91 women underwent TOT procedure by the same surgeon who used a PVDF tape and applied the modified outside-in technique. However, 59 patients had to be excluded from analysis. That is, 41 patients (45.1%) had an incomplete outcome and sonographic data; furthermore, 18 patients (19.8%) had incomplete sonographic data. Therefore, a total of 32 patients (35.2%) were finally included in the analysis. Baseline characteristics are presented in Table 1.

Measurements of the position of the TOT demonstrated ICC values of 0.85, 0.91, and 0.93 for slice number, TUD, and TSD, respectively (Table 2). These results indicate excellent agreement.

Between day one postoperatively and follow-up examination, the slice number where the tape was found did not change significantly as shown in Table 3. Furthermore, neither the TUD nor the TSD varied significantly (Table 3).

Cured and uncured patients did not differ in regard to age, vaginal delivery, and concomitant procedures. The follow-up time was significantly shorter in uncured patients (Table 1).

As shown in Table 4, the slice number where the tape was found differed significantly between cured and uncured patients. In particular, at the postoperative control the median slice number in cured patients was 7 (range, 5–8) compared to 6 (range, 5–7) in uncured patients. At the follow-up control, the median slice number was 7 (range, 5–8) and 5.5 (range, 4–7) in cured and uncured patients, respectively. Hence, cured patients were characterized by a tape position that was more caudal, compared to uncured patients.

In contrast, there were no remarkable differences regarding the TUD or TSD for both time points as seen in Table 4.

We selected age, postoperative TUD, and postoperative slice number as covariates, whereas subjective cure was chosen as the dependent parameter of the logistic regression. As the result of the stepwise backward regression method, a tape positioned in a more caudal slice increased the probability of subjective cure by a factor of 4.2 (95% CI 1.04–17.00, *P* = 0.04) (Table 5). This holds for the range, namely, from slices 5 to 8, measured in our study group.

## 4. Discussion

To our knowledge, this is the first study that describes the use of TUI for determining the position of the TOT. There are several studies analyzing the association between the position of the tape and the outcome in stress incontinent patients [2–9]. Most works measure the tape-symphysis pubis distance [2, 11, 12] on the midsagittal plane and, in addition, the tape position in relation to urethral length [2, 4–8, 11].

Our method simply requires counting the slices and search on the axial plane where the tape is seen dorsal to the urethra. As a major advantage, this approach is both fast and accurate, since it allows for an exact measurement of distances on the axial plane. TUI does not depend on provided single slice volumes [15] but allows the storage for later evaluation and the observation from any perspective. Our results demonstrate that our approach seems to be a reliable method with a high degree of accuracy as demonstrated by ICC values between 0.85 and 0.93.

No significant differences were found between sonographic parameters at postoperative and at follow-up control which may indicate a negligible displacement of the tape over a median period of time of 321 days. Similarly, other authors found no tape displacement in TOT patients, but only in patients with tension-free vaginal tapes (TVT) after a 6-month period [9]. Considering TVT, Kociszewski et al. [7] found a ventrocranial movement of the tape over a 4-year period, whereas other authors [16] observed a minor caudal migration (1.7 mm over a 3-year period). Similar observations were presented by Dietz et al. [12, 17]. However, in our study the patients were not controlled after the same time period (Table 1). That is, there was a significant shorter follow-up interval in uncured patients. Therefore, future research is needed that more systematically addresses to which extent different measurement intervals affect the results obtained by the present study.

In cured patients, the tape was typically placed at a more distal slice, most frequently at slice number 7, which is located 24 mm below the MUI. Assuming a urethral length of 40 mm, these results correspond to related findings that determine the lower middle and distal section as an optimal target position of the tape [2, 4, 8, 18]. Since our measurement technique does not consider the urethral length, a direct comparison with these works cannot be conducted, though.

In this study, we did not find an association between outcome and the TUD. Recent studies have shown that a distance smaller than 2 mm or larger than 5 mm correlates with* de novo* urgency symptoms and voiding problems [8]. In our cohort, most tapes were implanted within the recommended range [8] of the tape-urethra distance (postoperative: 0.45 ± 0.11, follow-up: 0.43 ± 0.12) in both cured and uncured patient groups. This could be a reason for the observation that the TUD did not correlate with outcome in our study. Furthermore, in our study the outcome and the TSD did not correlate. Other works [3, 11] report on better outcome given a smaller distance between tape and symphysis pubis. In our study, these distances are stable at 2 cm (postoperative: 2.09 ± 0.23; follow-up: 2.00 ± 0.35) similarly as in [2].

Different types of surgery techniques, follow-up time, and types of tapes are, among others, factors that hinder a direct comparison of the literature and might explain the diversity of published results [1].

Most surgeons use polypropylene as tape material. However, we selected PVDF tapes that consist of a monofilament sling with an excellent biocompatibility, thereby resulting in minimal foreign body reaction and optimal ingrowth [19, 20].

TOT, and in particular PVDF tapes [19], show no shift or only a negligible one during straining [9, 19, 21]. Hence, we considered it sufficient to perform the sonographic measurements while the patients were at rest.

Our study has further inherent limitations. We did not exclude concomitant surgery. However, the cases were equally distributed across the outcome groups. Furthermore, we applied a new incision for each surgery to repair the anterior or posterior vaginal wall. However, the incision is located in a more cranial position than the tape and should not interfere with the latter [22].

Same as other authors [2], we selected subjective definitions as outcome parameter, which delivers information about the patient's satisfaction over time.

More objective tests, such as stress test and urodynamic testing, provide only instantaneous information [2].

Furthermore, our dropout rate was rather high. Patients with incomplete outcome or sonographic data were excluded. Incomplete sonographic data reflects cases where data, either postoperative or at follow-up, was not found in our database. The reasons are generally not known due to the retrospective nature of the study. Partly, we assume errors during the storage of the data. In some cases, patients left the hospital before having conducted the postoperative ultrasound. In a significant number of cases, patients did not show up for a (recommended) follow-up examination despite our efforts of contacting them.

As our goal was to have a homogenous group of patients, we excluded those patients for whom either postoperative or follow-up data was missing.

Finally, the number of included patients is rather low. Therefore, we regard the present study as a pilot study. Nevertheless, the fact that we obtained significant differences in the slice number between uncured and cured patients despite the small sample size argues against a low statistical power of our study. As future work, prospective studies are planned to confirm these results.

## Figures and Tables

**Table 1:** Baseline characteristics of the study group and compared between the two outcome groups. Stress urinary incontinence (SUI). The results are given as counter (percentage) or median (range). ^*∗*^Fisher's exact or Mann-Whitney *U* test as appropriate, two-sided *P* value < 0.05 as significant.

Parameter	Total (*n* = 32)	Uncured from SUI (*n* = 6)	Cured from SUI (*n* = 26)	*P* value^*∗*^
Age	55 (34–81)	54 (37–77)	57 (34–81)	ns
Rectocele	4 (12.5)	1 (16.7)	3 (11.5)	ns
Anterior wall prolapse	9 (28.1)	2 (33.3)	7 (26.9)	ns
Descensus uteri	4 (12.5)	1 (16.7)	3 (11.5)	ns
Nullipara	2 (6.3)	0 (0.0)	2 (9.1)	ns
Concomitant surgery	6 (18.8)	1 (16.7)	5 (28)	ns
Follow-up time	321 (101–1905)	225 (51–370)	453 (123–1905)	0.04

**Table 2:** Intraclass correlation coefficient (ICC) (two-way random, single measurements, and absolute agreement). 95% confidence interval (CI). Distance between the anterior margin of the TOT and the inferoposterior margin of symphysis pubis (TSD). Distance between the anterior margin of the TOT and the longitudinal smooth muscle (LSM) complex of the urethra (TUD).

Parameter	ICC	95% CI	*P* value
Slice number	0.85	0.75–0.91	<0.001
TSD	0.91	0.84–0.95	<0.001
TUD	0.93	0.88–0.96	<0.001

**Table 3:** Comparison of sonographic parameters between postoperative and follow-up examinations. The results are given as median (range). Distance between the anterior margin of the TOT and the inferoposterior margin of the symphysis pubis (TSD). Distance between the anterior margin of the TOT and the longitudinal smooth muscle (LSM) complex of the urethra (TUD). ^*∗*^Wilcoxon-signed-rank test.

	Postoperative	Follow-up	*P* value^*∗*^
Slice number	7 (5–8)	7 (4–8)	0.11
TSD	2.09 (1.64–2.50)	2.0 (1.15–2.60)	0.30
TUD	0.46 (0.24–0.74)	0.43 (0.26–0.74)	0.22

**Table 4:** Comparison of sonographic parameters between cured and uncured patients. The results are given as median (range). Distance between the anterior margin of the TOT and the inferoposterior margin of the symphysis pubis (TSD). Distance between the anterior margin of the TOT and the longitudinal smooth muscle (LSM) complex of the urethra (TUD). ^*∗*^Mann-Whitney *U* test.

Outcome	Uncured (*n* = 6)	Cured (*n* = 26)	*P* value^*∗*^
*Postoperative*			
Slice number	6 (5–7)	7 (5–8)	0.05
TSD	1.9 (1.76–2.35)	2.1 (1.64–2.50)	0.33
TUD	0.41 (0.24–0.51)	0.47 (0.28–0.74)	0.17
*Follow-up*			
Slice number	5.5 (4–7)	7 (5–8)	0.05
TSD	1.8 (1.49–2.24)	2.03 (1.15–2.60)	0.14
TUD	0.41 (0.34–0.46)	0.43 (0.26–0.76)	0.94

**Table 5:** Results of the stepwise backward logistic regression, odds ratio (OR), and distance between the anterior margin of the TOT and the longitudinal smooth muscle (LSM) complex of the urethra (TUD).

	Age		TUD		Slice number	
	OR	*P* value	OR	*P* value	OR	*P* value
Step 1	1.01	0.851	48.18	0.46	3.51	0.09
Step 2			52.63	0.45	3.58	0.08
Step 3					4.20	0.04
